# Surface properties and biocompatibility of nanostructured TiO_2_ film deposited by RF magnetron sputtering

**DOI:** 10.1186/s11671-015-0732-7

**Published:** 2015-02-11

**Authors:** Asif Majeed, Jie He, Lingrui Jiao, Xiaoxia Zhong, Zhengming Sheng

**Affiliations:** Key Laboratory for Laser Plasmas (Ministry of Education) and State Key Laboratory of Advanced Optical Communication Systems and Networks, Department of Physics and Astronomy, Shanghai Jiao Tong University, Shanghai, 200240 China; Department of Physics, University of Azad Jammu & Kashmir, Muzaffarabad, A.K Pakistan; Department of Oral and Maxillofacial-Head and Neck Oncology, Shanghai 9th People’s Hospital, Shanghai Jiao Tong University School of Medicine, Shanghai, 200011 China

**Keywords:** TiO_2_, Working pressure, Substrate bias, Roughness, Wettability, Magnetron sputtering, Cell culturing

## Abstract

Nanostructured TiO_2_ films are deposited on a silicon substrate using 150-W power from the RF magnetron sputtering at working pressures of 3 to 5 Pa, with no substrate bias, and at 3 Pa with a substrate bias of −50 V. X-ray diffraction (XRD) analysis reveals that TiO_2_ films deposited on unbiased as well as biased substrates are all amorphous. Surface properties such as surface roughness and wettability of TiO_2_ films, grown in a plasma environment, under biased and unbiased substrate conditions are reported according to the said parameters of RF power and the working pressures. Primary rat osteoblasts (MC3T3-E1) cells have been cultured on nanostructured TiO_2_ films fabricated at different conditions of substrate bias and working pressures. The effects of roughness and hydrophilicity of nanostructured TiO_2_ films on cell density and cell spreading have been discussed.

**Electronic supplementary material:**

The online version of this article (doi:10.1186/s11671-015-0732-7) contains supplementary material, which is available to authorized users.

## Background

The use of biomaterials dates far back to ancient civilizations. Artificial eyes, ears, teeth, and noses were found on Egyptian mummies [1,2]. Waxes, glues, and tissues for the restoration of the missing or malfunctioning parts of the body were used by the Chinese and Indians. Over the centuries, developments in synthetic materials, surgical techniques, and sterilization methods have permitted the use of biomaterials in many ways [2,3]. Nowadays, a large number of devices and implants are used as a medical practice. Biomaterials in the form of implants (ligaments, vascular grafts, heart valves, intraocular lenses, dental implants, etc.) and medical devices (pacemakers, biosensors, artificial hearts, etc.) are extensively used to replace and/or restore the function of disturbed or deteriorated tissues or organs, and thus improve the quality of life of the patients.

Commercially pure titanium (CP-Ti) and its alloys are chosen and extensively used as biomaterials, i.e., for dental and orthopedic implants or prosthesis, because of their better corrosion resistance, lower modulus, superior biocompatibility, durability, and strength [2,4,5]. However, being bioinert, it is assumed that the integration of such implants in bone is not good enough. The biocompatibility of titanium used as an implant material is accredited to surface oxides naturally formed in air and/or physiological fluids [6,7]. The surface properties, including composition, roughness, hydrophilicity, texture, and morphology of the oxide on titanium, greatly influence the cellular behaviors, e.g., adhesion, morphologic change, functional alteration, proliferation, and differentiation [4,7-9]. Among surface properties, surface roughness and composition have been considered the most essential parameters for altering cell activity [10]. Titanium and its alloys, owing to their lower elastic modulus, are widely used as hard tissue replacements in artificial bones, joints, and dental implants, which, in general, are regarded as a biomechanical advantage on account of their smaller elastic modulus that can result in a smaller stress shielding [5,11].

The purpose of this work is to study the *in vitro* behavior of osteoblast cells cultured on nanostructured TiO_2_ film and investigate the effect of the nanostructured surface of TiO_2_ film on osteoblast cell density and cell spreading. Such accelerated cell density and cell spreading are beneficial for faster cure of dental and orthopedic patients, as well as for a variety of biomedical diagnostic and therapeutic applications [12].

Over the past few decades, implant coating has documented a wide range of applications. Thin-film coating of implant surfaces has been studied by several methods, including plasma spraying, dipping, electro-chemical deposition, pulsed laser deposition, ion beam dynamic mixing, and ion beam deposition [13]. Some of these methods have severe limitations such as poor adhesion, microcrack formation and phase changes at high temperature, non-uniformity, and improper microstructural control, all of which make them inadequate for implant systems [14,15].

Most recently, magnetron sputtering deposition has been proposed by many researchers as a flexible deposition technique that offers many advantages including high-deposition rates; ease of sputtering any metal, alloy or compound; the formation of high-purity films; extremely high adhesion to films; and the ability to form dense coatings [16-18].

In this letter, we report the effect of working pressure and substrate bias on the surface roughness of a TiO_2_ film deposited by RF magnetron sputtering and see the influence of surface roughness in nanoscale [19] on surface hydrophilicity and cell behavior over the surface.

## Methods

### Fabrication of nanostructured TiO_2_ films

**Figure 1:**
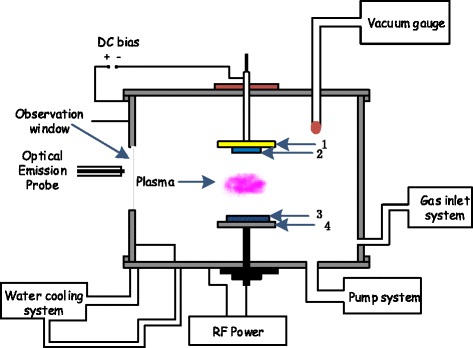
**Schematic of RF magnetron sputtering: 1) substrate stage, 2) glass substrates, 3) Ti targets, and 4) RF-powered electrode.**

The TiO_2_ films were deposited by using RF magnetron sputtering system (JGP-450 A, China), whose schematic diagram is shown in the Figure 1. For the deposition of thin films, silicon and titanium were used as the substrate and target materials, respectively. The purity of the titanium target fixed on the magnetron cathode was 99.9% with a diameter of 60 mm (Beijing General Research Institute for Non-Ferrous Metals, China). The silicon substrate (100) with a diameter of 25.4 mm was mounted on the substrate stage separated at a distance of 70 mm from the titanium target. Before mounting the silicon substrate onto the substrate stage in the vacuum chamber, it was cleaned ultrasonically in acetone followed by ethanol and finally in deionized water for 15 min in each solution and dried thereafter. A pumping system comprising of mechanical and the turbo-molecular pumps was implied to achieve the base pressure down to 1.4 × 10^−3^ Pa inside the chamber. In order to remove the residual gases, the chamber was heated along with the pumping. Argon and oxygen, as sputtering and reactive gases, with a purity of 99.99% and 99.95%, respectively, were introduced into the chamber one after another and controlled by standard mass flow controllers (Beijing Jianzhong Machine Co. Ltd., China). The gas pressure was adjusted with the help of a throttle valve. The ZDF-2 AK compound vacuum meter (Beijing Xinhua Vacuum Instrument Factory, China) was used to monitor the vacuum pressure in the chamber. Prior to thin-film deposition, Ti ceramic target was pre-sputtered in an argon environment for 10 min by RF power supply working at a frequency of 13.56 MHz to weed out the surface adsorptions and contaminations. The effect of working pressures of 3 and 5 Pa, on the surface morphology of the films at an RF power of 150 W, without bias was studied by depositing TiO_2_ films on silicon substrates for 5 h in each case. Also, a sample at a working pressure of 3 Pa with a bias of −50 V at an RF power of 150 W for 5 h was prepared/deposited so as to compare the surface morphologies of the films with and without bias. The substrate temperature was measured with a thermocouple in contact with the surface of the substrate. The flow rates utilized for argon and oxygen were staying constant, that is 30 and 10 sccm (i.e., standard cubic centimeter per minute), respectively, during the whole experiment. The summary of the deposition condition is given in Table 1.

**Table 1:** **Summary of deposition conditions of TiO**_**2**_ **films**

Base pressure	1.4 ×10^−3^ Pa
Working pressures	3 Pa at a substrate bias of 0 V,
	5 Pa at a substrate bias of 0 V,
	3 Pa at a substrate bias of −50 V
Deposition time	5 Hrs for each sample
RF Power	150 W
Argon flow rate	30 sccm
Oxygen flow rate	10 sccm
Target to substrate distance	70 mm
Diameter of silicon (100) substrate	25.4 mm
Diameter of the titanium target	60 mm

### Surface characterization

The phase characterization of the TiO_2_ films deposited on silicon substrates at different working pressures with RF power of 150 W, with and without bias, was carried out by X-ray diffraction (XRD), using CuK_α_ radiation (*λ* = 0.154056 nm) for 2*θ* values ranging from 20° to 80°. The diffractometer (XRD, Rigaku D/max 2550 VB/PC, Rigaku, Tokyo, Japan) was operated at 40 kV and 200 mA with a scanning speed of 8°/min at 2*θ* steps of 0.020°. The angle of the incident beam was 0.9°. The surface topography of the said TiO_2_ films, deposited under the same conditions, was characterized by atomic force microscopy (AFM, Nanoscope 3A, DI, USA) and the root-mean-square (RMS) roughness was estimated by an image analysis software called Nanoscope®III. The wettability of the films' surface was observed through water contact angle measurements, using contact angle measurement equipment (OCA 20, Dataphysics, Germany).

### Cell culture

Primary rat osteoblasts (MC3T3-E1) were cultured for 7 days in a humidified atmosphere of 5% CO_2_ –95% air at 37°C in 25 cm^2^ flasks until confluent. Cells were then detached using trypsin/EDTA (0.25% *w*/*v* trypsin/0.02% EDTA of pH 7.2). Subsequently, cells were re-suspended in the supplemented culture medium as described above and seeded with the density of 2 × 10^4^ cells/cm^2^ on the specimen surfaces for the biocompatibility study. After being fixed in 4% paraformaldehyde (Sigma, USA), cells were stained with acridine orange (AO, Sigma, USA), and the cells' behavior for 12 h over the specimen surface was examined with a fluorescent microscope (×200).

## Results and discussions

### X-Ray diffraction analysis

**Figure 2:**
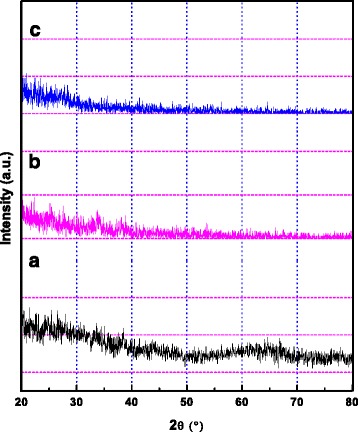
**XRD patterns of TiO**_**2**_ **samples at an RF power of 150 W at working pressures of (a) 3 Pa and (b) 5 Pa, without bias, and (c) 3 Pa at a substrate bias of −50 V.**

XRD patterns, for TiO_2_ films deposited for 5 h on silicon substrates at working pressures of (3, 5) Pa without substrate bias and at 3 Pa with bias (−50 V), using an RF power supply with a 13.56-MHz frequency and maximum 150 W power used to excite the plasma, are obtained as shown in Figure 2a,b,c. These XRD patterns reveal that TiO_2_ films deposited on silicon substrates are all amorphous in nature, as they do not show any characteristic peak of TiO_2_, as shown in Figure 2a,b,c. It is necessary for crystallization that the deposition particles of TiO_2_ have enough energy to diffuse. Therefore, amorphous structures of TiO_2_ thin films can be attributed to low-surface mobility of deposition particles, and hence, the deposition particles do not possess enough energy to crystallize [20]. The working pressure at which the sputtering takes place has an important impact on the growth process of thin films. At high working pressures, such as (3, 5) Pa as shown respectively in Figures 2 a,b, because of shorter collisional mean free path of the particles, the sputtered atoms suffer multiple collisions before reaching the substrate, so they cannot make their proper arrangement on reaching the substrate on account of their reduced kinetic/collisional energies and hence form large clusters of particles during the film deposition [21]. High kinetic energies are needed for the reorganization of atoms on the substrate which leads to an improved crystalline structure. Therefore, thin films grown at high working pressures are usually more rough and amorphous [22-24]. One of the most important deposition parameter that influences the structure of thin films is the substrate temperature. If the substrate is not heated intentionally (it reaches about 100°C only because of sputtering and the thermal flow from plasma [25]) [26], the films with amorphous structures are normally produced. This can be explained by the fact that the adatom mobility is negligible so that the atom condensation takes place near the point of impingement. Therefore, it is inferred that the deposited films are generally amorphous if the substrate is not heated during the deposition and the distance between the substrate and target is large [4].

When a negative bias is applied to the substrate, it can lead to an increase in the energy of the surface atoms resulting in an enhanced surface diffusion, which can cause better adhesion, nucleation, and crystal structure [27]. When the substrate is biased to −50 V, more energy is transferred from ions driven by the substrate bias to the growing film, which can make the film more compact. Nevertheless, the transferred energy is not high enough to make the film crystallinized; thus, the TiO_2_ film, obtained under the substrate bias condition, shows an amorphous structure [28].

### Surface features

**Figure 3:**
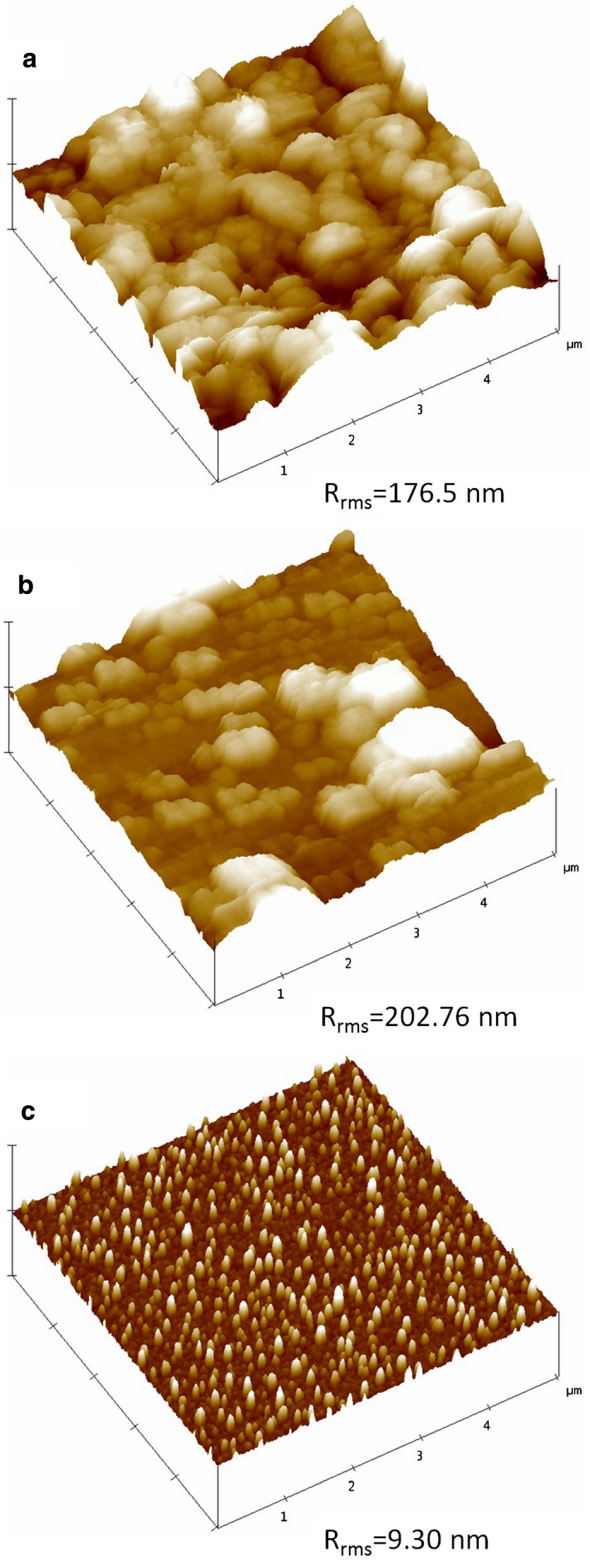
**AFM images of titanium dioxide layer deposition by RF magnetron sputtering at (a) 3 Pa and (b) 5 Pa, without substrate bias, and (c) 3 Pa with a substrate bias of −50 V.**

The AFM images of surface structure of TiO_2_ films deposited by RF magnetron sputtering at a constant power of 150 W and at different working pressures, i.e., (3, 5) Pa, have been shown in Figure 2a,b for unbiased substrates. Both the surface structures exhibit the porous configuration. The results revealed that at working pressures of 3 and 5 Pa, the films deposited on unbiased substrates possessed very high surface roughness with some valleys and almost spherical agglomerates. The roughness of these thin films was characterized according to RMS roughness value. In our experiment, the RMS roughness values were estimated to be 176.50 and 202.76 nm for the thin films grown at 3 and 5 Pa, respectively, for the unbiased substrate. At high working pressures, sputtered atoms or clusters reached the substrate with reduced kinetic energies, which caused the surface roughness to increase [22,23]. The employing of substrate bias to the film at a working pressure of 3 Pa modified the surface morphology; as seen, the agglomerates grew up along the surface but collapsed in the vertical direction. Along with the disappearance of the so-called profile valleys, the RMS roughness showed an obvious reduction from 176.50 to 9.30 nm as the substrate bias (V) changed from 0 to −50 V, as shown in Figure 3c. The surface-smoothening effect was mainly attributed to the ion bombardment for which the arriving adatoms could diffuse more freely onto the film surface [29].

### Surface wettability analysis

Wettability is a solid–liquid-vapour interfacial phenomenon and is characterized by measuring the water contact angle formed between a liquid drop and a solid surface [30]. When an interface exists between a liquid and a solid, the angle between the surface of the liquid and the outline of the contact surface is described as the contact angle θ (lower case theta). Water contact angle measurement for characterizing surface wettability is assumed to be a relatively simple, useful, and sensitive tool for assessing hydrophobicity or hydrophilicity of a surface, surface heterogeneity, surface roughness, solid surface energy, liquid surface tension and line tension [31]. The contact angle can characterize the wettability of the surface of a solid by a liquid, i.e. the interaction between a solid and a liquid surface at the interface, and represents a thermodynamic relationship known as the Young equation [6], which relates the angle $\uptheta$ to the interfacial solid–vapour (SV), solid–liquid (SL) and liquid–vapour (LV) free energies ($\gamma$) as follows:

1 $$ {\gamma}_{SV}={\gamma}_{SL}+{\gamma}_{LV} \cos \uptheta $$

This theoretical relation is true only for ideally smooth and homogeneous solid surfaces.

Wettability of surfaces is not only influenced by the liquid chemical properties but can also be strongly affected by surface roughness. In 1936, Wenzel reported that the roughness of a homogenous solid surface affects contact angle measurements (referred to as apparent angles) as follows:

2 $$ cos{\theta}_A= rcos\theta $$

where *θ*A is an apparent contact angle and *r* is the ratio of the real rough surface area to the projected perfectly smooth surface, also referred to as the Wenzel factor which in other words is proportional to the extension of surface area due to roughness. It should be noted that for rough surface *r* > 1 and for perfectly smooth surface *r* = 1, and therefore, cos$\theta$A *= cos*$\theta$, where $\theta$ is the contact angle corresponding to the ideal smooth surface also called equilibrium (Young) angle. In practice, this theory is used for contact angle 0° < $\theta$< 90° [32,33].

**Figure 4:**
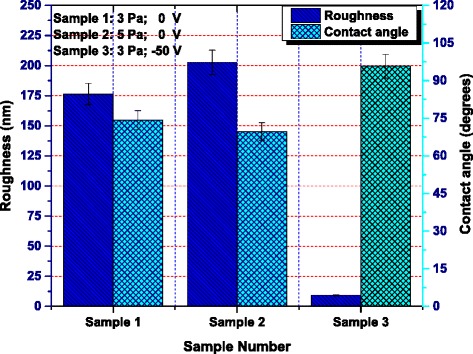
**Magnitude of RMS roughness and static water contact angles for each sample.** Sample 1, *P* = 3 Pa, 0 V; sample 2, *P* = 5 Pa, 0 V; sample 3, *P* = 3 Pa, −50 V.

The variations in water contact angle for the samples deposited at 3 and 5 Pa without substrate bias and also at 3 Pa with bias (−50 V), in static mode, together with their RMS roughness are shown in Figure 4. The static water contact angle for the samples deposited at 3 and 5 Pa without substrate bias was found to be varying from 74.3° to 69.70° when the RMS roughness value was increased from 176.50 to 202.76 nm. The static water contact angle (at varying bias conditions of the substrate from 0 to −50 V) for the samples deposited at 3 Pa was found to be altering from 74.30° to 95.73°_,_ when the RMS roughness value was decreased from 176.50 to 9.301 nm.

For more authenticity and reliability of the results, the contact angle measurements were also carried out dynamically. If the three-phase contact line is in actual motion, the contact angle produced is called a ‘dynamic’ contact angle. In particular, the contact angles formed by expanding and contracting the liquid are referred to as the advancing contact angle $\theta$a and the receding contact angle $\theta$r, respectively.

**Figure 5:**
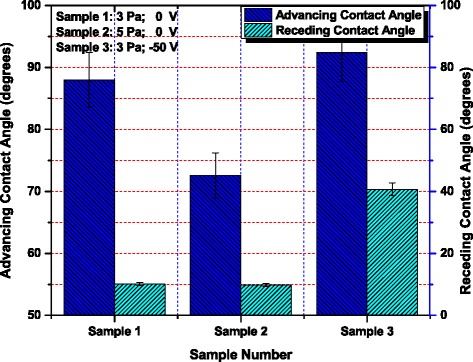
**Magnitude of dynamic advancing contact angles and the corresponding receding contact angles for each sample.** Sample 1, *P* = 3 Pa, 0 V; sample 2, *P* = 5 Pa, 0 V; sample 3, *P* = 3 Pa, −50 V.

The advancing contact angles for samples 1, 2, and 3 have been estimated to be 88.0°, 72.6°, and 92.4°, respectively, while the corresponding receding contact angles have been measured to be 10.1°, 9.8°, and 40.7°, respectively, as illustrated by Figure 5. The contact angle hystereses for samples 1, 2, and 3, determined from their corresponding advancing and receding angles, are 77.90, 62.80, and 51.70, respectively.

Usually the hysteresis is greater for the rough surfaces, but it is dominated by chemical interactions and heterogeneities rather than roughness itself [32]. Nonetheless, to the best of our knowledge and studies, no one has reported the effect of contact angle hysteresis on the cell behavior over surface. So further investigation is needed to analyze the contact angle hysteresis on the cell behavior over the surface.

The results might be well elaborated in terms of surface energy, which could play a very vital role on the surface wettability, and the surfaces with high surface energy (greater surface roughness) are usually regarded as hydrophilic. According to the illustration of the film deposition at different working pressures, a higher working pressure leads to a rougher surface with larger surface defects and greater surface energy, which results in a more hydrophilic surface as shown in Figure 4. Moreover, Figures 4 and 5 reveal the comparison of static as well as the dynamic (both advancing and receding) contact angles, indicating that the corresponding static as well as dynamic angles are showing the same increasing/decreasing trend. This clearly justifies that the experimental results are well consistent with each other.

### Cell density and cell spreading on TiO_2_ samples

**Figure 6:**
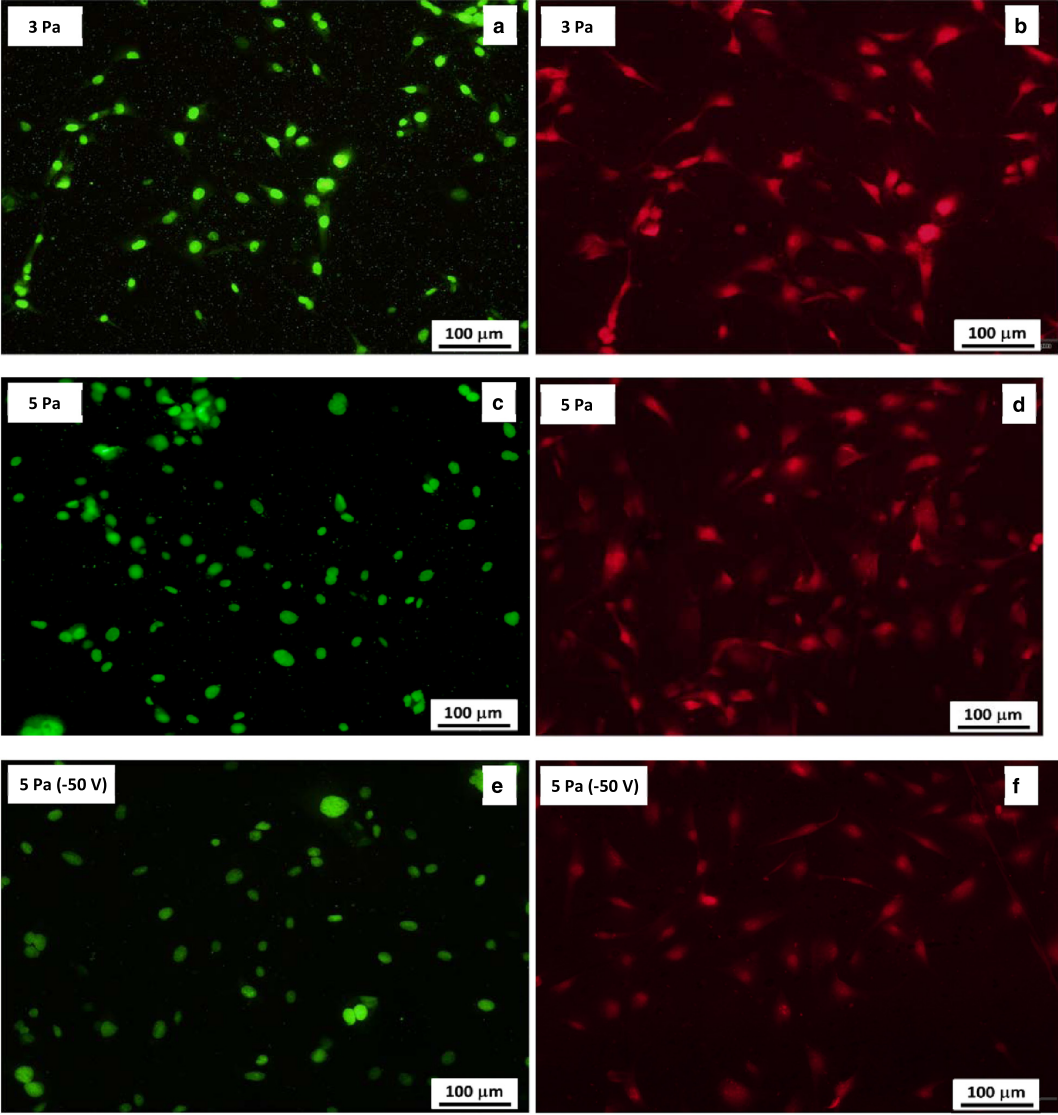
**Images of primary rat osteoblasts (MC3T3-E1) on TiO**_**2**_ **films.** Images of primary rat osteoblasts (MC3T3-E1) on TiO_2_ films deposited at **(a, b)** 3 Pa, **(c, d)** 5 Pa, without biasing the substrate, and **(e, f)** 3 Pa at a substrate bias of −50 V.

The microscopic view of studying the morphology of PRO (MC3T3-E1) cells cultured on TiO_2_ samples prepared at(3, 5) Pa (no substrate bias) and 3 Pa (biased at −50 V) are shown in Figure 6. The cells on these samples are cultured for 12 h and are named as sample 1, 2, and 3, respectively. Having cultured the cells, there have been three kinds of cells attached onto the surface of each specimen/sample as shown in Figure 6. i) Not spread: cells are still spherical, protrusions are not yet produced; ii) partially spread: cells began to spread laterally with at one or more sides; and iii) fully spread: at this stage, the extensions of plasma membrane are completely confluent [6].

**Figure 7:**
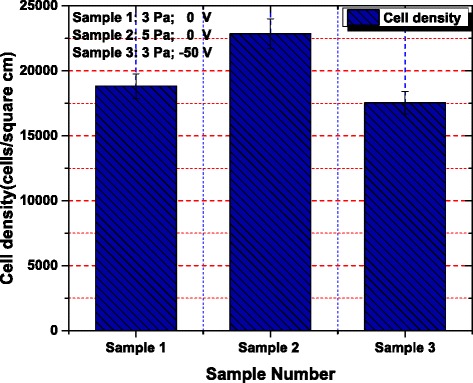
**Variation of cell density for each sample.** Sample 1, *P* = 3 Pa, 0 V; sample 2, *P* = 5 Pa, 0 V; sample 3, *P* = 3 Pa, −50 V.

For the determination of the cell density of the primary rat osteoblasts (MC3T3-E1) cells, cultured on each sample, an Image Pro Plus software was utilized. The average cell density on sample 1 was estimated to be 18,826.27 cell/cm^2^, on sample 2 was about 22,861.92 cell/cm^2^, and on sample 3, it was about 17,537.26 cell/cm^2^. The analysis showed that the magnitude of the cell density on sample 2 was the highest and that of the sample 3 was the least among the three samples as represented by the bar graph in Figure 7. It has also been shown in Figure 6 that the percentage of spread cells among the attached cells is higher on samples 1 and 2 than that of sample 3. Among the above stated three samples, the percentage of cell spreading on sample 2 is the highest and that of sample 3 is the lowest. The result revealed that the cell spreading somehow had the dependence on surface properties such as surface roughness and the surface wettability. Considering the surface roughness and the wettability properties of different specimens/samples together as given in Figure 4, it was predicted that cells easily spread on a hydrophilic surface and the surface energy played an important role in cell spreading.

The number of adherent cells was determined by means of nuclei quantification on the TiO_2_-nano, Ti-nano, and Ti-micro substrates [34]. The interaction between the outermost surface of a biomaterial and its environment was a highly dynamic process, in which direct and indirect cell adhesions induced by protein previously adsorbed onto the surface were two competing processes. In general, the direct cell adhesion tends to occur efficiently on hydrophilic surfaces but inefficiently on hydrophobic surfaces, whereas indirect cell adhesion dominates over smooth and hydrophobic surfaces [35].

In comparison with samples 1 and 3, sample 1 with a rough surface promotes direct cell adhesion; on the contrary, sample 3 with a smooth surface encourages indirect cell adhesion through adsorption of proteins on its surface. That is probably the main reason that there is no big difference in cell densities over the samples 1 and 3. As regards sample 1 and 2, both of them having rough surfaces are dominated by direct cell adhesion, since sample 2 is rougher and more hydrophilic than sample 1, so cell density over sample 2 is relatively higher.

On the other hand, most of the studies [36,37] indicate that the cell tends to spread on the hydrophilic surfaces rather than hydrophobic surfaces, which is consistent with our experiment. More studies are still underway to investigate the effect of surface roughness and wettability on the biocompatibility of the TiO_2_ films including cell adhesion, proliferation, and differentiation.

## Conclusions

In this article, TiO_2_ films, with different surface roughnesses measured in nanometer scale, were deposited on a silicon substrate using a power level of 150 W from the RF magnetron sputtering at different working pressures under varying bias conditions for biocompatibility analysis. The XRD analysis of the TiO_2_ film revealed its amorphous nature. Water contact angle measurements clarified that the rough surface was more hydrophilic than the smooth surface, which was well elaborated in terms of surface energy that could play a very vital role on the surface wettability.

It was concluded that the surface could be designed to influence cell density and cell spreading, and RF reactive magnetron sputtering method could be a potential method to improve the cytocompatibility of titanium-based implants by depositing a layer of TiO_2_ with suitable roughness.
